# Myocardial work abnormalities in rheumatoid arthritis with preserved left ventricular ejection fraction are more closely related to disease activity than to disease duration: a prospective cross-sectional study

**DOI:** 10.3389/fmed.2026.1872016

**Published:** 2026-07-06

**Authors:** Xiaolong Yu, Yanjia Lu, Jing Xi, Mingsi Wang, Junli Chen, Ruixiao Song

**Affiliations:** 1 Department of Ultrasonics, Wujin Hospital Affiliated with Jiangsu University, Changzhou, Jiangsu, China; 2 Science and Education Section, The Wujin Clinical College of Xuzhou Medical University, Changzhou, Jiangsu, China; 3 Center of Interventional Radiology and Vascular Surgery, Nurturing Center of Jiangsu Province for State Laboratory of AI Imaging & Interventional Radiology, Department of Radiology, Zhongda Hospital, Medical School, Southeast University, Nanjing, China; 4 Department of Ultrasound, Zhongda Hospital, Medical School, Southeast University, Nanjing, China; 5 Department of Ultrasound, Nanjing First Hospital, Nanjing Medical University, Nanjing, China; 6 Laboratory of Cough, Affiliated Kunshan Hospital of Jiangsu University, Suzhou, China; 7 Department of Pulmonary and Critical Care Medicine, The First Affiliated Hospital of Soochow University, Suzhou, Jiangsu, China

**Keywords:** disease activity, myocardial work, pressure-strain loop, rheumatoid arthritis, subclinical left ventricular dysfunction

## Abstract

**Objective:**

To investigate subclinical left ventricular (LV) dysfunction in rheumatoid arthritis (RA) patients with preserved left ventricular ejection fraction (pLVEF) using myocardial work (MW) analysis, and to determine if myocardial damage is closely associated with disease duration.

**Patients and methods:**

We prospectively enrolled 59 RA patients with preserved LVEF between January 2021 and September 2023 at Wujin Hospital Affiliated to Jiangsu University. The RA cohort was stratified by disease duration into a short-duration group (≤10 years, *n* = 28) and a long-duration group (>10 years, *n* = 31), and 35 healthy controls. LV function was assessed using left ventricular pressure-strain loop (LV-PSL) technology to derive global longitudinal strain (GLS) and MW parameters, including global work index (GWI), global constructive work (GCW), global wasted work (GWW), and global work efficiency (GWE). Disease activity was quantified using the Disease Activity Score in 28 joints based on C-reactive protein (DAS28-CRP).

**Results:**

Compared to controls, both RA groups demonstrated significantly impaired myocardial mechanics, with reduced absolute values of GLS, GWI, and GCW (all *p* < 0.05). GCW was significantly reduced in the long-duration group (*p* < 0.05), while the short-duration group showed a borderline decreasing trend. Crucially, GWE was negatively correlated with inflammatory activity (DAS28-CRP: *r* = −0.369, *p* = 0.004). However, no significant correlation was found between any MW parameter and disease duration (all *p* > 0.05).

**Conclusion:**

In RA patients, subclinical myocardial dysfunction is detectable across disease durations, characterized by impaired myocardial work. This dysfunction is associated with current disease activity rather than cumulative disease duration. MW analysis offers a sensitive tool for identifying subclinical myocardial work impairment in RA, and should be considered for incorporation into proactive cardiovascular risk screening protocols for RA patients.

## 1 Introduction

Rheumatoid arthritis (RA), the most common systemic autoimmune disease, is increasingly recognized for its cardiovascular (CV) complications, which have become major determinants of long-term prognosis (1). According to the European Society of Cardiology (ESC) guidelines, the risk of CV disease in patients with RA is approximately twofold higher than in the general population, and this excess risk emerges early in the disease course (2). Notably, RA is associated with an increased risk of heart failure. However, because early myocardial injury is often subclinical, conventional assessments frequently fail to detect it in a timely manner, leading to missed opportunities for optimal intervention (3, 4).

Notably, accumulating epidemiological and clinical evidence suggests that RA confers a disproportionate risk of heart failure with preserved ejection fraction (HFpEF) compared with the general population and even with other inflammatory conditions (5). Unlike heart failure with reduced ejection fraction, HFpEF is characterized by diastolic dysfunction, impaired myocardial relaxation, and subclinical systolic abnormalities that are undetectable by conventional LVEF measurement. RA patients with preserved LVEF thus represent a high-priority population for early myocardial surveillance (6), since standard echocardiographic assessment may substantially underestimate their true cardiovascular risk. LV-PSL technology, by providing a load-compensated, integrative assessment of myocardial mechanics, is uniquely positioned to identify this at-risk population before overt HFpEF develops (7).

Systemic, persistent inflammation is considered a central mechanism in CV involvement in RA. Emerging evidence shows that proinflammatory cytokines such as tumor necrosis factor-*α* (TNF-α), interleukin-1β (IL-1β), and interleukin-6 (IL-6) not only mediate joint destruction but also act directly on cardiomyocytes. IL-6 and TNF-α have been independently associated with higher coronary artery calcium scores and increased CV risk, supporting the hypothesis that RA-related CV risk is closely linked to elevated cytokine levels and their deleterious effects on the endothelium (8, 9). Mechanistically, these cytokines activate multiple signaling pathways—including JAK/STAT3, MAPK, and NF-κB—promoting cardiomyocyte apoptosis, myocardial fibrosis, vascular dysfunction, and direct impairment of myocardial contractility (10).

Large clinical studies have, however, emphasized another dimension: disease duration itself as a key determinant of CV risk in RA. In a long-term cohort of 571 Japanese patients with RA (mean follow-up 11.7 years), those who experienced CV events had significantly longer disease duration than those without events (15.0 ± 12.7 vs. 10.8 ± 9.7 years, *p* = 0.01). Cox proportional hazards analysis identified longer disease duration as an independent risk factor for CV disease (hazard ratio 1.57, 95% CI 1.09–2.30, *p* = 0.02) (11). In line with cumulative exposure hypotheses, another study demonstrated that RA disease duration correlated positively with carotid–femoral pulse wave velocity (*r* = 0.340, *p* < 0.001), with patients having disease duration ≥10 years exhibiting greater arterial stiffness than those with duration <2 years—suggesting an “accelerated vascular aging” effect of long-standing RA (12). These data support the concept that chronic inflammatory exposure progressively impairs endothelial function, facilitating atherogenesis and plaque destabilization.

That said, the notion of disease duration as an independent risk factor is not universally accepted. One large cohort study reported that, after adjustment for disease activity, disease duration did not appear to independently influence cardiovascular disease risk, and the risk did not further increase in patients with RA whose disease duration exceeded 10 years (13). This view posits that current inflammatory activity, rather than cumulative duration, is the primary driver of CV risk.

Thus, a central question remains unresolved: Are myocardial functional abnormalities in RA primarily determined by cumulative disease duration or by current inflammatory activity? Studies supporting the primacy of inflammatory activity show that short-term CV risk tracks with disease activity and that anti-inflammatory therapy significantly reduces CV events (14). This ongoing debate has direct implications for clinical risk stratification: Should CV risk assessment prioritize current inflammation control or cumulative disease exposure?

Longitudinal evidence from the CADERA study further supports the primacy of inflammatory activity over disease duration: reduced aortic distensibility - a measure of central vascular stiffness - was observed in early RA patients and improved with effective anti-inflammatory treatment (15). Strikingly, when the same cohort was reassessed 11 years later under good inflammatory control, aortic distensibility showed no significant change over time (16), implying that sustained disease control may attenuate or halt progressive cardiovascular deterioration. These data are consistent with the hypothesis that active systemic inflammation, rather than the passage of time per se, drives vascular and potentially myocardial functional changes in RA.

Left ventricular ejection fraction (LVEF) remains the primary echocardiographic index of systolic function, yet it has well-recognized limitations for detecting subtle contractile dysfunction. Recent advances in echocardiographic imaging have substantially improved myocardial functional assessment. In particular, noninvasive LV-PSL analysis has shown unique value in patients with preserved LVEF. LV-PSL quantifies myocardial work (MW) by integrating two-dimensional speckle-tracking echocardiography (2D-STE)–derived global longitudinal strain (GLS) with noninvasive arterial pressure, thereby mitigating after load dependency and providing a novel dimension for evaluating myocardial mechanics (17, 18). In the LV-PSL analysis, the area enclosed by the pressure–strain loop corresponds to the Global Work Index (GWI), reflecting the total mechanical work executed by the left ventricular myocardium throughout the cardiac cycle, specifically from mitral valve closure to mitral valve opening (19). Therefore, left ventricular pressure–strain loop (LV-PSL) serves as a more comprehensive tool for assessing myocardial function, capable of measuring cardiac motion and evaluating work efficiency. With advances in echocardiographic technology and computational capability, noninvasive LV-PSL has been widely adopted and is particularly powerful for detecting subclinical myocardial dysfunction in patients with preserved EF (20–22).

Building on our recent studies using noninvasive LV-PSL analysis - which have demonstrated the clinical value of myocardial work indices in detecting subclinical dysfunction in patients with preserved LVEF (23) and shown that global work efficiency is moderately associated with disease activity in RA patients (DAS28-CRP) (24) - an important question remains unresolved: to what extent is subclinical myocardial dysfunction in RA closely associated with cumulative disease duration versus current disease activity. While our earlier work in coronary artery disease validated the load-adjusted sensitivity of LV-PSL–derived parameters (23) and our recent RA cohort study (24) focused exclusively on disease activity stratification, neither study addressed the relative contribution of disease duration to myocardial dysfunction. Importantly, the utility of myocardial work analysis has been independently validated in other autoimmune diseases, including systemic sclerosis and systemic lupus erythematosus, where similar subclinical myocardial dysfunction was detected despite preserved LVEF (25, 26). Furthermore, cardiac magnetic resonance studies have independently confirmed immuno-inflammatory myocardial remodeling in RA, characterized by elevated native T1 values and reduced GLS (27). In the previous RA study, patients were analyzed as a single cohort stratified only by disease activity and were not categorized according to disease duration, precluding a direct assessment of duration-related effects on myocardial work.

Therefore, in the present prospective study, we employed advanced noninvasive LV-PSL analysis to assess myocardial work in RA patients with preserved LVEF and explicitly compared myocardial work parameters among patients with different disease durations. By relating these indices to both disease duration and current disease activity, we aimed to delineate the relative contributions of cumulative disease burden and ongoing inflammatory activity to subclinical myocardial dysfunction. We hypothesized that myocardial work abnormalities would be detectable across disease duration strata - including in patients with relatively shorter disease duration - and would be more significantly associated with current disease activity than with disease duration perse. Clarifying these relationships is expected to provide new evidence informing the ongoing debate on “duration-driven” versus “inflammation-driven” cardiovascular risk in RA and to contribute to a more refined framework for cardiovascular risk stratification and early cardiac monitoring in this population.

## 2 Methods

### 2.1 Study population

We prospectively enrolled 59 patients with RA and preserved LVEF who presented to Wujin Hospital, Affiliated to Jiangsu University, between January 2021 and September 2023. Patients were stratified by disease duration into a long-duration group (>10 years; *n* = 31) and a short-duration group (≤10 years; *n* = 28). This cutoff was selected in accordance with EULAR cardiovascular risk management guidelines, which identify disease duration exceeding 10 years as a significant factor augmenting cardiovascular risk (2). However, to maximize statistical power and explore linear relationships, disease duration was primarily analyzed as a continuous variable. The long-duration group comprised 31 patients (5 men, 26 women; median age 59 years [IQR 52, 71.25]); the short-duration group comprised 28 patients (9 men, 19 women; median age 63 years [IQR 53.25, 71.75]). Thirty-five age-appropriate healthy individuals undergoing routine health examinations during the same period served as controls (11 men, 24 women; median age 68 years [IQR 60, 74]). Disease activity in the RA cohort was assessed using the Disease Activity Score in 28 joints based on C-reactive protein (DAS28-CRP) (28). *Inclusion criteria*: ① Sinus rhythm. ② Diagnosis of RA according to the 2010 American College of Rheumatology/European League Against Rheumatism (ACR/EULAR) classification criteria (29). ③ No obvious regional wall motion abnormalities on conventional echocardiography and preserved LVEF (≥50%). *Exclusion criteria*: To minimize confounding hemodynamic factors and isolate the specific impact of RA-related inflammation on myocardial mechanics, stringent exclusion criteria were applied. Subjects were excluded if they presented with: ① Cardiovascular comorbidities: Cardiac arrhythmias, clinical heart failure, cardiomyopathy, known significant coronary artery disease (≥50% stenosis on coronary angiography or history of coronary revascularization), prior myocardial infarction, congenital heart disease, significant valvular heart disease, or left ventricular hypertrophy secondary to longstanding hypertension. Cardiac arrhythmias, defined as any non-sinus rhythm identified on a standard 12-lead electrocardiogram at the time of enrolment, including atrial fibrillation, atrial flutter, second- or third-degree atrioventricular block, and sustained ventricular tachycardia; patients with known paroxysmal arrhythmias (e.g., paroxysmal atrial fibrillation) documented on prior Holter monitoring were also excluded. ② Systemic and other conditions: Renal dysfunction, defined as an estimated glomerular filtration rate (eGFR) < 60 mL/min/1.73m^2^ or a known diagnosis of chronic kidney disease (CKD) stage 3 or above; coexisting connective tissue diseases other than RA; history of malignancy; or exposure to known cardiotoxic chemotherapy agents. ③ Technical limitations: Suboptimal echocardiographic image quality hindering accurate strain analysis. This rigorous selection process was designed to reduce inter-individual variability.

The diagnostic criteria for diabetes mellitus were based on the 1999 World Health Organization (WHO) guidelines (30): fasting blood glucose ≥7.0 mmol/L or 2-h blood glucose ≥11.1 mmol/L after oral glucose tolerance test (OGTT). Hypertension was defined according to the International Society of Hypertension (ISH) 2020 Global Hypertension Practice Guidelines (31): systolic blood pressure ≥140 mmHg and/or diastolic blood pressure ≥90 mmHg. Smoking was defined following standardized WHO definitions as smoking more than one cigarette per day for at least 6 consecutive or cumulative months.

No participants had missing data for the primary echocardiographic outcome variables; participants with suboptimal image quality precluding accurate strain analysis were excluded prior to final enrolment, as specified in the exclusion criteria.

The study protocol conforms to the ethical guidelines of the 1975 Declaration of Helsinki as reflected in *a priori* approval by the Ethics Committee of Wujin Hospital Affiliated with Jiangsu University.(Approval No. 2021-NT-036). Written informed consent was obtained from all participants prior to enrollment.

### 2.2 Instruments and methods

#### 2.2.1 Equipment

A GE Vivid E95 ultrasound diagnostic system equipped with an M5Sc-D phased array probe (frequency range 1.7–3.3 MHz) was used. An EchoPAC workstation was utilized for image post-processing.

#### 2.2.2 Image acquisition

Blood pressure was measured in a resting state 10 min prior to echocardiographic examination using an upper-arm cuff. Subjects were examined in the left lateral decubitus position, with electrocardiographic monitoring to ensure a stable ECG tracing. Routine transthoracic echocardiography was performed using the GE Vivid E95 with an M5Sc-D probe; the frame rate was maintained between 50 and 80 frames per second.

According to American Society of Echocardiography guidelines (32), parasternal long-axis views of the left ventricle were obtained to measure the left ventricular end-diastolic diameter (LVEDD), interventricular septal thickness at end-diastole (IVSD), and left ventricular posterior wall thickness at end-diastole (LVPWD). The biplane Simpson’s method was used to calculate left ventricular end-diastolic volume (LVEDV), left ventricular end-systolic volume (LVESV), and LVEF.

Subjects were instructed to hold their breath while dynamic two-dimensional images were acquired from the apical four-chamber, two-chamber, and long-axis views, recording at least three stable and clear cardiac cycles for each view. In the apical long-axis view, the mitral and aortic valves were visualized, and aortic valve pulsed Doppler spectral tracings were recorded to define end-systolic time points. All cine loops and images were stored for subsequent analysis.

#### 2.2.3 Image analysis

Systolic blood pressure measured immediately prior to echocardiography was entered into the EchoPAC workstation. The Event Timing function was used to determine the opening and closing times of the aortic and mitral valves, based on the spectral Doppler tracings. Analysis began with the apical three-chamber view. The optimal cardiac cycle was selected using the “Cycle” function, followed by clicking “APLAX” for automatic tracking of the endocardial and epicardial borders. Manual adjustment was performed if the automated tracking was suboptimal, after which “Approve and select next” was selected. The same approach was applied to the apical four-chamber and two-chamber views. A 17-segment model was used. The software calculated GLS as a weighted average of segmental peak systolic longitudinal strain values, generating strain curves and bull’s-eye plots for storage. Patients with suboptimal tracking in one or more myocardial segments were excluded from the study.

**Figure 1:**
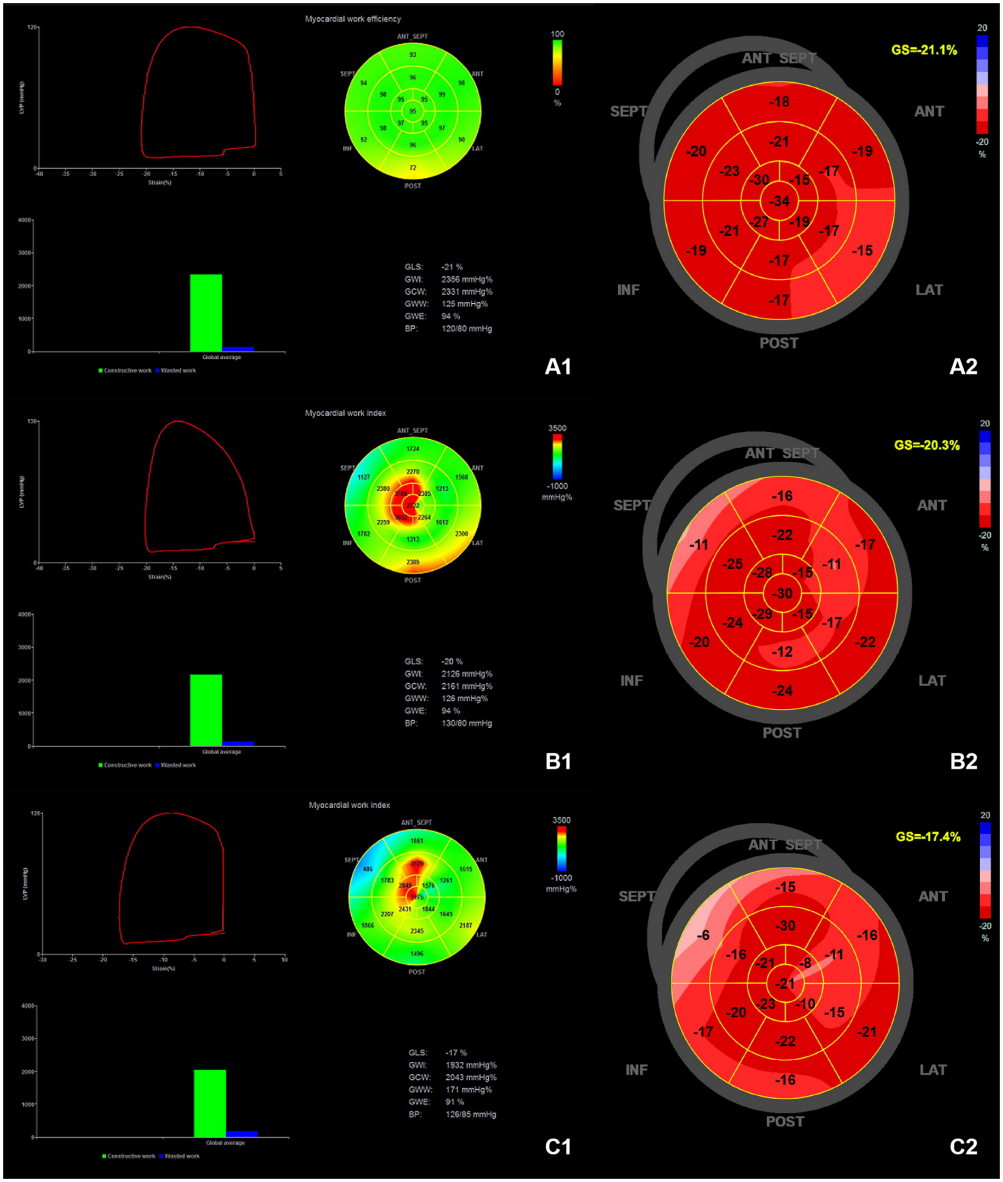
LV-PSL evaluation of left ventricular MW parameters. **(A1)** An estimated pressure-strain loop and the Bull’s eye of the regional distribution of MW parameters from a healthy volunteer in the control group. **(A2)** Left ventricular systolic longitudinal strain and Bull’s eye plot from the same healthy volunteer. **(B1)** An estimated pressure-strain loops and the Bull′s eye of the regional distribution of MW parameters from a patient in the long-duration RA group. **(B2)** Left ventricular systolic longitudinal strain and Bull’s eye plot from the same patient. **(C1)** An estimated pressure-strain loops and the Bull′s eye of the regional distribution of MW parameters from a patient in the short-duration RA group. **(C2)** Left ventricular systolic longitudinal strain and Bull′s eye plot from the same patient.

Post-processing was conducted using the “Myocardial Work” analysis package to generate bull’s-eye plots, following the protocol detailed in our previous study (23). The following left ventricular MW parameters were quantified: (1) Global Constructive Work (GCW): The work contributing to left ventricular ejection, performed by myocardial shortening during isovolumic contraction and systole as well as myocardial lengthening during isovolumic relaxation. (2) Global Wasted Work (GWW): The energy expenditure that does not contribute to effective ejection, i.e., myocardial lengthening during systole and shortening during isovolumic relaxation. (3) Global Work Efficiency (GWE): Calculated as GCW / (GCW + GWW) × 100%. (4) Global Work Index (GWI): The weighted average myocardial work index of the 17 segments, reflecting overall myocardial work during the cardiac cycle (Figure 1).

### 2.3 Statistical analysis

Statistical analyses were performed using SPSS version 26.0 (IBM Corp., Armonk, NY, USA) and MedCalc (MedCalc Software, Ostend, Belgium). The distribution of continuous variables was assessed using the Kolmogorov–Smirnov test. Normally distributed data are presented as mean ± standard deviation (x̄ ± s) and were compared among the three groups using one-way analysis of variance (ANOVA). Post-hoc pairwise comparisons for normally distributed variables were performed using Tukey’s Honest Significant Difference (HSD) test to control for family-wise error rate. Non-normally distributed data are presented as median (interquartile range, IQR) and were compared using the Kruskal–Wallis test, with pairwise comparisons adjusted using the Bonferroni correction.

Categorical variables are presented as counts (*n*) and were compared using the chi-square (χ^2^) test. Spearman’s rank correlation was used to evaluate associations between MW parameters and disease duration, DAS28-CRP, GLS, and LVEF.

All tests were two-sided, with a significance level of *α* = 0.05. Correlation strength was interpreted as follows: strong, *r* ≥ 0.60; moderate, 0.40 ≤ *r* < 0.60; weak, *r* < 0.40.

To validate the reliability of echocardiographic measurements, inter- and intra-observer reproducibility were assessed in a randomly selected subset of 20 subjects from both the control and RA groups. Intra-observer variability was evaluated by the same observer repeating the measurements after 2 weeks. Inter-observer variability was determined by a second independent observer blinded to clinical details and original measurements. Agreement was quantified using the Intraclass Correlation Coefficient (ICC) with a two-way mixed model for intra-observer and a two-way random model for inter-observer assessment. Bland–Altman analyses were also performed to visualize the agreement.

A post-hoc power analysis was conducted using G*Power software (Version 3.1.9.7). For the one-way ANOVA comparing GWI among the three study groups (*N* = 94), with an observed effect size f of 0.35 and *α* = 0.05, the achieved statistical power (1-*β*) was 0.857, exceeding the conventional 0.80 threshold. We note that post-hoc power analyses have recognized methodological limitations as indices of sample adequacy, and this result should be interpreted as descriptive of the observed effect size rather than as confirmatory evidence of sufficient sample size. The relatively small sample size remains a limitation of this study (see Limitations section), and replication in larger cohorts is warranted.

Given the exploratory-confirmatory hybrid design of this study, correlation analyses were structured hierarchically. The association between GWE and DAS28-CRP was defined as the primary outcome correlation *a priori*, based on our preceding cohort study and central hypothesis, and was evaluated without adjustment for multiplicity. All secondary correlations were regarded as hypothesis-generating; these remained statistically significant after false discovery rate correction using the Benjamini-Hochberg procedure at a 5% threshold.

## 3 Results

### 3.1 Baseline characteristics of the study population

A total of 94 participants were included, comprising 59 patients with RA - 31 with long disease duration and 28 with short disease duration - and 35 healthy controls. Baseline characteristics were well balanced across groups. There were no significant differences in demographic or clinical variables, including age, sex, systolic blood pressure, diastolic blood pressure, and the prevalence of hypertension or diabetes (all *p* > 0.05) (Table 1).

**Table 1:** Baseline characteristics of the study population.

Item	Long-duration group	Short-duration group	Control group	*t/Z*	*P*
Number	31	28	35	/	/
Male [*n* (%)]	5 (16.1%)	9 (32.1%)	11 (31.4%)	2.600	0.273
Age, years [*M* (P25, P75)]	59 (52, 71.25)	63 (53.25, 71.75)	68 (60, 74)	5.120	0.077
Systolic blood pressure	123 (120, 128.5)	125 (120, 127.5)	126 (122, 130)	3.701	0.181
Diastolic blood pressure	80 (70, 85.5)	77 (70, 80)	83 (75, 85)	2.940	0.230
Hypertension [*n* (%)]	10 (28.6%)	6 (21.4%)	12 (38.7%)	2.140	0.343
Diabetes [*n* (%)]	4 (12.9%)	3 (10.7%)	8 (22.9%)	2.032	0.362

### 3.2 Between-group differences in laboratory inflammatory markers

Laboratory findings reflected the typical inflammatory activity observed in RA. Compared with healthy controls, RA patients exhibited significantly higher levels of multiple inflammatory biomarkers, including rheumatoid factor (RF), C-reactive protein (CRP), erythrocyte sedimentation rate (ESR), and neutrophil percentage (NEUT) (all *p* < 0.05). Subgroup analyses further showed no significant differences in inflammatory markers between the short-duration group (disease duration ≤10 years) and the long-duration group (disease duration >10 years) (all *p* > 0.05) (Table 2).

**Table 2:** Between-group differences in laboratory inflammatory markers.

Item	Long-duration group	Short-duration group	Control group	*t/Z*	*p*
Number	31	28	35	/	/
RF (IU/mL)	170 (56.3, 404)^a^	67.45 (35.77, 254.75)^a^	11.0 (7.2, 15.65)	40.864	<0.001
CRP (mg/L)	21.3 (5, 80)	9.04 (2.51, 35.8)	2.4 (1.6, 4.1)	3.141	0.028
ESR (mm/H)	66 (36, 104)^a^	57 (35, 82.75)^a^	2.6 (2.0, 4.3)	45.002	<0.001
Neutrophil	3.99 (2.85, 5.79)^a^	3.97 (3.1, 5.3)	3.14 (2.28, 3.94)	8.996	0.011
Lymphocyte	1.52 (1.09, 1.87)	1.48 (1.25, 1.97)	1.46 (1.29, 1.61)	1.881	0.390
Platelet	290 (213, 333)	252 (214.25.2, 310.5)	239 (205, 273)	2.267	0.322
DAS28-CRP	4.31 (2.88, 5.61)	3.775 (3.1, 5.05)	/	/	/

RF, rheumatoid factor; CRP, C-reactive protein; ESR, Erythrocyte sedimentation rate; DAS28-CRP, Disease Activity Score in 28 joints based on C-reactive protein. ^a^Compared with the control group *p* < 0.05.

### 3.3 Comparative analysis of cardiac structural and functional parameters and myocardial work parameters

#### 3.3.1 Conventional echocardiographic parameters

This study enrolled RA patients with pLVEF. There were no significant differences among the RA subgroups and the control group with respect to LVEF, IVSD, LVEDV, LVESV, and heart rate (HR) (all *p* > 0.05). Conventional echocardiography did not detect early myocardial dysfunction in RA patients (Table 3).

**Table 3:** Comparative analysis of cardiac structural and functional parameters and myocardial work parameters.

Item	Long-duration group	Short-duration group	Control group	*t*/Z	*p*
Number	31	28	35	/	/
GWI	2120.96 ± 314.99*	2165.25 ± 405.76*	2393.28 ± 335.15	5.515	0.005
GCW	2251.61 ± 297.44*	2284.46 ± 435.10	2480.85 ± 340.15	4.621	0.012
GWW	196 (150, 266)	193 (102, 215.75)	164.5 (118.5, 211.25)	1.223	0.542
GWE (%)	91 (88, 93)	92.5 (90, 94)	93 (91, 94)	3.274	0.195
GLS	−19.42 (−20.38, −17.56)*	−20.43 (−22, −18.53) *	−21.8 (−22.5, −20)	19.746	<0.001
PSD	45.96 (42, 73)	48.44 (37.08, 57.86)	40.8 (35.25, 53.6)	2.051	0.359
LVEF (%)	65.49 (59.9, 68.1)	65.83 (61.42, 68.25)	64 (59.25, 72.5)	0.818	0.664
IVSD (mm)	8.1 (7.4, 9.1)	7.25 (7.0, 8.25)	7.65 (7.1, 8.65)	4.986	0.083
LVEDV (mL)	96.4 (90.3, 114.80)	94.2 (73.55, 103.5)	92.2 (73.4, 109. 8)	1.529	0.446
LVESV (mL)	33.5 (28, 45)	29.45 (23.75, 38.65)	29.55 (22.6, 42.25)	2.430	0.297
HR	76 (70, 91)	75.5 (71.25, 89.5)	78 (67.5, 83)	0.927	0.629

GWI, global work index; GCW, global constructive work; GWW, global wasted work; GWE, global work efficiency; GLS, global longitudinal strain; PSD, Peak strain dispersion; LVEF, left ventricular ejection fraction; IVSD, interventricular septal thickness at end-diastole; LVEDV, left ventricular end-diastolic volume; LVESV, left ventricular end-systolic volume. *Compared with the control group *p* < 0.05.

#### 3.3.2 Detailed assessment of myocardial strain and myocardial work parameters

**Figure 2:**
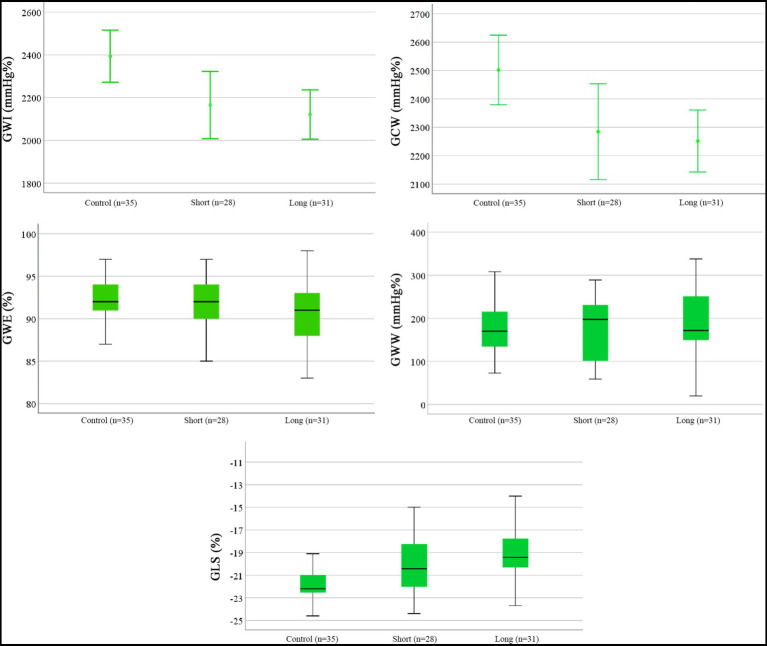
Detailed assessment of myocardial strain and myocardial work parameters. Myocardial strain parameters and myocardial work parameters among the control, short -duration and long-duration groups. From the control group to the short and long duration groups, GWI, GCW, and GWE decreased sequentially. GWI, global work index; GCW, global constructive work; GWW, global wasted work; GWE, global work efficiency; GLS, global longitudinal strain.

Using pressure-strain loop (PSL) technology, a significant reduction was observed in the absolute value of GLS [−19.42 (−20.38, −17.56)% and −20.43 (−22.00, −18.53)% vs. − 21.8 (−22.5, −20.0)%, all *p* < 0.05] and GWI [2120.96 ± 314.99 mmHg% and 2165.25 ± 405.76 mmHg% vs. 2393.28 ± 335.15 mmHg%, all *p* < 0.05] in both the long-duration and short-duration RA groups compared with controls. Regarding GCW, a significant reduction was observed in the long-duration group compared with controls [2251.61 ± 297.44 mmHg% vs. 2480.85 ± 340.15 mmHg%, *p* < 0.05]; however, the short-duration group showed a decreasing trend that approached but did not reach statistical significance (2284.46 ± 435.10 mmHg%, *p* = 0.058). Compared with controls, there was a numerical stepwise decline in myocardial work parameters with increasing disease duration, but differences between the long-duration and short-duration RA groups were not statistically significant. GWW showed a gradual increase with longer disease duration, but this trend was not statistically significant (*p* > 0.05) (Table 3; Figure 2).

##### 3.3.2.1 Correlation with GLS

**Figure 3:**
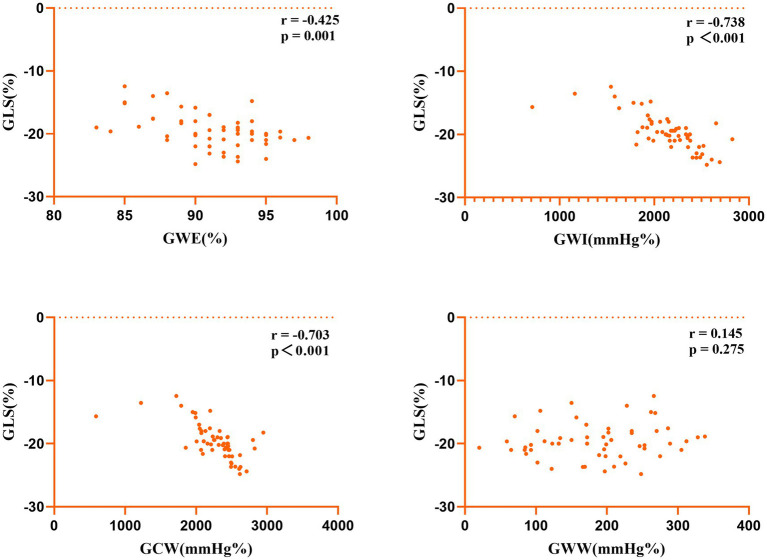
The correlations of myocardial work parameters with GLS in the RA groups. GWE, GWI, and GCW in the RA groups had moderate to strong negative correlations with GLS, while GWW showed no significant correlation with GLS. GWI, global work index; GCW, global constructive work; GWW, global wasted work; GWE, global work efficiency; GLS, global longitudinal strain.

GWI showed a strong negative correlation with GLS (*r* = −0.738, *p* < 0.001), as did GCW (*r* = −0.703, *p* < 0.001), while GWE exhibited a moderate negative correlation with GLS (*r* = −0.425, *p* = 0.001) (Figure 3). Since GLS is expressed as a negative value, with greater absolute values indicating better systolic function, these inverse correlations are physiologically consistent. In contrast, GWW was not significantly correlated with GLS (*r* = 0.145, *p* = 0.275), suggesting that wasted work primarily reflects mechanical dyssynchrony rather than overall myocardial deformation capacity.

##### 3.3.2.2 Reproducibility of myocardial work and strain parameters

The reliability analysis demonstrated excellent agreement for both intra- and inter-observer measurements. The ICCs for MW parameters and GLS ranged from 0.864 to 0.986, indicating high reproducibility. Specifically, the intra- and inter-observer ICCs for GWI were 0.986 (95% CI: 0.965–0.995) and 0.970 (95% CI: 0.753–0.992), respectively. For GLS, the intra- and inter-observer ICCs were 0.969 (95% CI: 0.925–0.988) and 0.958 (95% CI: 0.896–0.983). Detailed results are provided in Table 4 and Supplementary Figure 1.

**Table 4:** Intra- and inter-observer reproducibility of myocardial work indices and GLS.

Parameter	Intra-observer ICC (95% CI)	Inter-observer ICC (95% CI)
GWI	0.986 (0.965–0.995)	0.970 (0.753–0.992)
GCW	0.976 (0.940–0.990)	0.951 (0.639–0.986)
GWW	0.874 (0.665–0.951)	0.864 (0.603–0.950)
GWE	0.943 (0.862–0.977)	0.911 (0.790–0.964)
GLS	0.969 (0.925–0.988)	0.958 (0.896–0.983)

GWI, global work index; GCW, global constructive work; GWW, global wasted work; GWE, global work efficiency; GLS, global longitudinal strain.

##### 3.3.2.3 Correlation with LVEF

**Figure 4:**
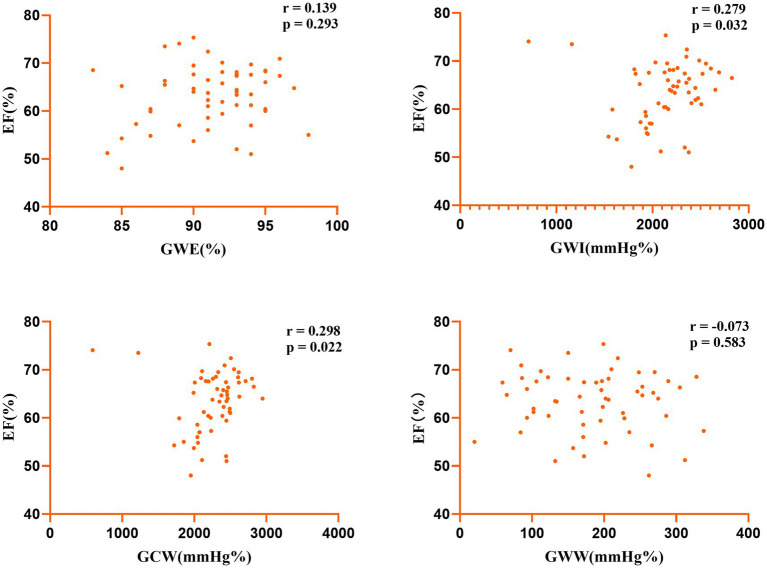
The correlations of myocardial work parameters with EF in the RA groups. None of the myocardial work parameters were significantly correlated with EF. GWI, global work index; GCW, global constructive work; GWW, global wasted work; GWE, global work efficiency; EF, ejection fraction.

None of the four myocardial work parameters (GWI, GCW, GWW, and GWE) were significantly correlated with LVEF (all *p* > 0.05) (Figure 4), which may be attributed to the fact that all enrolled participants had preserved ejection fraction within the normal range.

##### 3.3.2.4 Correlation with disease activity and duration

**Figure 5:**
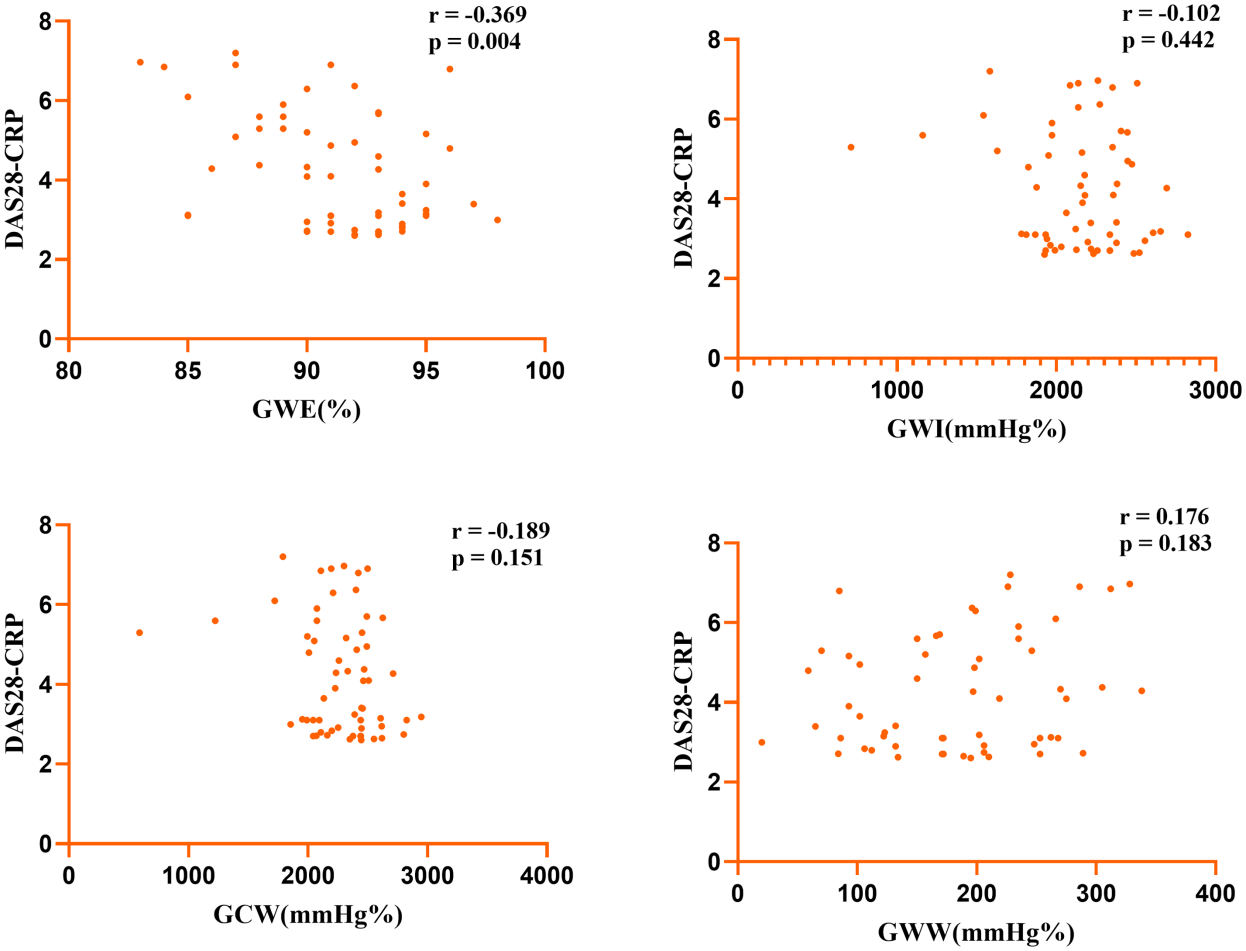
The correlations of myocardial work parameters with DAS28-CRP in the RA groups. GWE in the RA group exhibited a negative correlation with DAS28-CRP, while other myocardial work parameters showed no significant correlation with DAS28-CRP. GWI, global work index; GCW, global constructive work; GWW, global wasted work; GWE, global work efficiency; DAS28-CRP, Disease Activity Score in 28 joints based on C-reactive protein.

**Figure 6:**
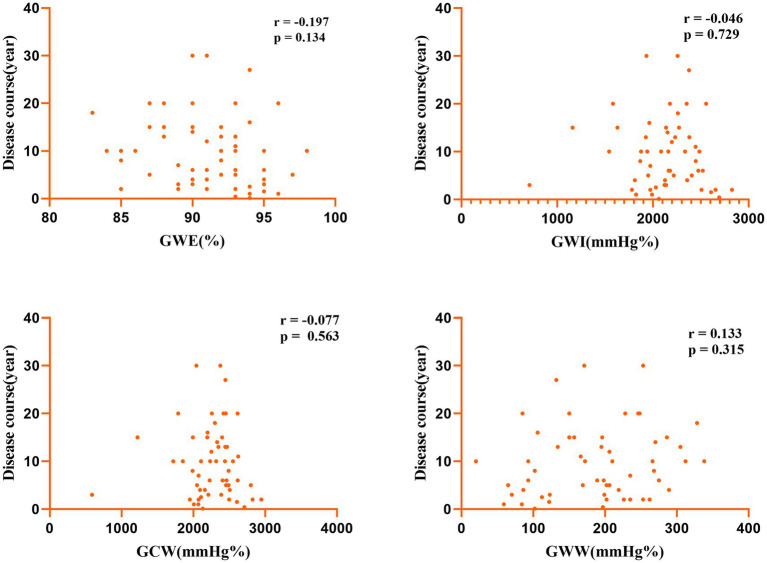
The correlations of myocardial work parameters with Disease duration in the RA groups. None of the myocardial work parameters were significantly correlated with Disease duration. GWI, global work index; GCW, global constructive work; GWW, global wasted work; GWE, global work efficiency.

GWE demonstrated a negative correlation with DAS28-CRP (*r* = −0.369, *p* = 0.004) (Figure 5), indicating that higher disease activity is associated with reduced myocardial work efficiency. In contrast, regarding disease duration, both statistical analysis and visual inspection of scatter plots (Figure 6) revealed no significant linear or non-linear associations with GWI, GCW, or GWE (all *p* > 0.05). These findings are consistent with myocardial work impairment being detectable across a range of disease durations - including in patients with relatively shorter disease duration-rather than a condition that emerges exclusively in long-standing disease. However, the absence of a significant duration - MW correlation should be interpreted cautiously, as it reflects only the absence of detectable linear progression within this cohort and does not constitute direct evidence of early onset. This highlights that current disease activity is more strongly associated with myocardial mechanics than disease chronicity itself. Additionally, GWW was not significantly correlated with GLS, LVEF, DAS28-CRP, or disease duration (all *p* > 0.05), supporting the notion that myocardial work abnormalities in RA primarily manifest as reductions in effective work and efficiency rather than an increase in wasted work.

##### 3.3.2.5 Multivariable analysis and interaction testing

To assess the independent association between disease activity and myocardial work efficiency, a multivariable linear regression model was constructed adjusting for age and systolic blood pressure (Table 5). DAS28-CRP remained significantly associated with GWE (*β* = −0.291, *p* = 0.017), independent of potential confounders. Furthermore, we evaluated the interaction between disease duration and DAS28-CRP (Table 6). The non-significant interaction term (*p* = 0.718) indicates that the association between DAS28-CRP and GWE does not vary by disease duration, consistent with the interpretation that current disease activity may be more closely associated with myocardial work efficiency than disease chronicity in this cohort.

**Table 5:** Multivariable linear regression for GWE.

Variable	Unstandardized B (95% CI)	Standardized β	*p*-value
(Constant)	105.900 (98.233, 113.568)	-	<0.001
DAS28-CRP	−0.646 (−1.170, −0.122)	−0.291	0.017
Age	−0.093 (−0.162, −0.024)	−0.321	0.009
SBP	−0.049 (−0.103, 0.006)	−0.208	0.079

**Table 6:** Multivariable linear regression analysis with interaction term for global work efficiency (GWE).

Variable	Unstandardized *β*	SE	Standardized β	*t*	*p*-value	95% CI
Constant	105.827	3.984	-	26.560	<0.001	97.828 to 113.826
DAS28-CRP	−0.497	0.506	−0.224	−0.982	0.331	−1.512 to 0.519
Disease duration (years)	0.025	0.145	0.063	0.170	0.866	−0.266 to 0.315
DAS28-CRP × Duration (Interaction)	−0.013	0.036	−0.148	−0.363	0.718	−0.086 to 0.060
Age (years)	−0.094	0.036	−0.323	−2.611	0.012	−0.166 to −0.022
Systolic BP (mmHg)	−0.050	0.028	−0.215	−1.810	0.076	−0.106 to 0.005

SE, standard error; CI, confidence interval; DAS28-CRP, disease activity score in 28 joints based on C-reactive protein; BP, blood pressure.

## 4 Discussion

This study employed LV-PSL technology to identify subclinical myocardial dysfunction in patients with RA and preserved LVEF and to explore the relationship between cardiac impairment and disease duration. Our findings revealed that MW indices were significantly reduced in RA patients - even in the presence of normal conventional echocardiographic parameters - and that these alterations were detectable across disease duration strata, showing no significant association with disease duration but a close relationship with disease activity. These results provide new insights into the mechanisms of RA-related cardiovascular involvement and underscore the need for proactive screening and timely clinical attention in all RA patients.

### 4.1 Mechanistic framework underlying myocardial work abnormalities in RA

The mechanistic pathways discussed in this section and in Section 4.3 are drawn primarily from preclinical, *in vitro*, and human non-RA studies. Because the present study did not incorporate cytokine profiling, these pathways cannot be directly tested by our data. They are presented to provide a pathophysiological framework that contextualizes our observational findings and supports their biological plausibility.

In this study, RA patients exhibited significantly elevated inflammatory markers (RF, CRP, ESR, and NEUT), accompanied by reductions in MW parameters, consistent with our previous research. We previously confirmed that myocardial work impairment occurs in RA patients with preserved EF and correlates strongly with disease activity (24). Chronic systemic inflammation—the hallmark of RA—is central to this process. Proinflammatory cytokines such as TNF-*α*, IL-1β, and IL-6 not only mediate joint destruction but also directly damage cardiomyocytes, leading to fibrosis, apoptosis, and contractile dysfunction (33, 34). Where human clinical RA data are available, these are explicitly identified. In a human prospective RA cohort study, Alexandre et al. (35) reported that persistent proinflammatory activation markedly increases the risk of myocardial dysfunction and heart failure in RA. Based on experimental and mechanistic evidence, cytokines have been shown to impair myocardial function through multiple pathways in preclinical models: (1) induction of cardiomyocyte apoptosis and interstitial fibrosis; (2) disruption of calcium-handling mechanisms, impairing excitation–contraction coupling; (3) promotion of oxidative stress and mitochondrial dysfunction; (4) activation of neurohormonal signaling contributing to maladaptive remodeling (36, 37).

### 4.2 Advantages of PSL technology

Subclinical left ventricular (LV) dysfunction in RA has been extensively investigated. In a seminal study published in 2012, Sitia et al. (38). demonstrated that 2D-STE could detect subclinical cardiac involvement in RA patients, with GLS serving as a sensitive marker for the early identification of myocardial impairment. More recently, Tarjanyi et al. (39) further confirmed that patients with RA exhibited significantly reduced LV GLS (22 ± 2% vs. 24 ± 3%; *p* < 0.01), accompanied by increased LV mass, thereby underscoring the value of strain parameters for the early detection of myocardial damage. Consistent with these findings, our study also revealed that the absolute value of GLS was significantly decreased in all RA case groups compared to controls [−19.42 (−20.38, −17.56); −20.43 (−22, −18.53) vs. −21.8 (−22.5, −20); *p* < 0.05]. In recent years, accumulating evidence has emphasized the clinical utility of speckle-tracking echocardiography (STE) in detecting myocardial ischemia in patients without overt wall motion abnormalities (40).

Although GLS remains a key index for LV systolic function, its diagnostic accuracy is limited by marked load dependence (41). In contrast, myocardial work parameters derived from LV-PSL technology integrate both strain and estimated LV pressure, effectively minimizing afterload effects and accurately reflecting the dynamic LV pressure–strain relationship (42). Zhong et al. (26) demonstrated that PSL-based myocardial work indices permit noninvasive evaluation of myocardial performance in systemic lupus erythematosus (SLE), with GWE outperforming GLS in detecting subclinical dysfunction.

Our study provides new insights by comprehensively evaluating myocardial work using LV-PSL technology in patients with RA and preserved LVEF. While Lang et al. (43) established the theoretical foundation and Russell et al. (19) confirmed clinical applicability, the clinical utility of LV-PSL technology has since been extended by multiple independent groups across various clinical contexts. For instance, Zhang et al. demonstrated that myocardial work parameters detect subclinical dysfunction in systemic sclerosis patients with normal GLS (25), and Zhong et al. reported similar findings in systemic lupus erythematosus (26). In the oncology setting, Guo et al. showed that myocardial work indices predict chemotherapy-induced cardiotoxicity earlier than GLS alone (44). Our research group has recently validated the diagnostic and prognostic utility of this methodology in other populations with preserved LVEF, including patients with stable coronary artery disease (23). In that study, we demonstrated that LV-PSL - derived myocardial work indices - particularly GWI - effectively detect subclinical myocardial dysfunction and correlate with disease severity as quantified by angiographic SYNTAX scores, even when conventional echocardiographic parameters remain normal. Building on that technical validation, the present study extends the application of LV-PSL technology to the field of autoimmune inflammatory disease, where data specific to RA with preserved LVEF remain scarce. We observed significantly reduced GWI in both short- and long-duration RA groups compared with controls. However, significant impairment in GCW was confined to the long-duration group, while the short-duration group exhibited a decreasing trend that approached statistical significance. Both parameters were strongly correlated with GLS (*r* = −0.738, *p* < 0.001 for GWI; *r* = −0.703, *p* < 0.001 for GCW). This novel use of PSL technology transcends conventional strain analysis by incorporating afterload, offering a comprehensive and physiologically accurate evaluation of myocardial efficiency in RA.

### 4.3 Relationship between disease duration and myocardial dysfunction

Our findings revealed a nuanced and clinically meaningful dual role of disease duration in RA-related myocardial dysfunction, extending beyond a linear relationship. Regardless of disease duration, RA patients showed evidence of myocardial injury. On the one hand, intergroup comparisons clearly demonstrated that cardiac impairment was present in RA patients regardless of disease duration. Both short-duration (≤10 years) and long-duration (>10 years) groups had significantly lower GWI compared with healthy controls. GCW was significantly lower in the long-duration group, while the short-duration group showed a decreasing trend that approached statistical significance (Table 3). This pattern aligns with robust epidemiological evidence indicating that RA confers a 1.5- to 2-fold increased risk of cardiovascular mortality and is an independent predictor of heart failure.

Paradoxically, while GWI, GCW, and GWE tended to decline with longer duration, differences between short- and long-duration RA subgroups were not statistically significant, and disease duration as a continuous variable was not linearly associated with any MW parameter (all *p* > 0.05; Table 7). These results are consistent with the view that myocardial work impairment in RA is not restricted to patients with long-standing disease, and may be detectable even among those with shorter - though not necessarily early - disease duration. This observation should be interpreted with appropriate caution: the ≤10-year subgroup cannot be equated with “early RA,” and the numerical trend toward greater MW impairment in the long-duration group (lower GWI, lower GCW, lower GLS absolute values) acknowledges that cumulative disease exposure may contribute to a gradual subclinical progression, even if this did not reach statistical significance in our sample. This observation contrasts with the findings of Sitia et al. (38) and Rudominer et al. (45) who reported cumulative disease duration as a major determinant of cardiac involvement in cross-sectional studies. The discrepancy may, at least in part, be attributed to methodological differences—previous studies typically analyzed RA patients as a single cohort compared with controls, whereas our analysis stratified patients according to disease duration (≤10 years vs. >10 years). The seemingly contradictory results underscore that the strongest predictor of impaired myocardial work efficiency may not be temporal disease duration per se, but rather current disease activity. Taken together with our previous LV-PSL studies—including a recent validation in patients with coronary artery disease and preserved LVEF, demonstrating that myocardial work parameters effectively detect subclinical dysfunction and correlate with angiographic disease severity (23), and our RA cohort study showing that GWE is moderately correlated with disease activity (24)—the present duration-stratified data are compatible with (or provide further associative support for) an inflammation-associated hypothesis for myocardial dysfunction in RA. Importantly, while our CAD study confirmed the technical reliability and load-adjusted sensitivity of LV-PSL methodology in detecting early myocardial impairment, the current investigation addresses a fundamentally different clinical question: whether myocardial work abnormalities in RA are more closely associated with cumulative disease duration or with current disease activity. In both our earlier RA cohort (24) and the present study, myocardial work abnormalities were consistently linked to measures of current disease activity, whereas in the current study no significant association was observed between myocardial work indices and disease duration. Although causality cannot be inferred from these observational designs, the converging evidence from two independent datasets suggests that the level of ongoing systemic inflammatory activity, rather than cumulative disease duration alone, may be more closely linked to subclinical myocardial work impairment in RA.

**Table 7:** Correlation between myocardial work parameters and clinical indicators.

	GLS		EF		DAS28-CRP		Disease duration	
	*r*	*p*	*r*	*p*	*r*	*p*	*r*	*p*
GWE	−0.425	0.001	0.139	0.293	−0.369	0.004	−0.197	0.134
GWI	−0.738	<0.001	0.279	0.032	−0.102	0.442	−0.046	0.729
GCW	−0.703	<0.001	0.298	0.022	−0.189	0.151	−0.077	0.563
GWW	0.145	0.275	−0.073	0.583	0.176	0.183	0.133	0.315

GWI, global work index; GCW, global constructive work; GWW, global wasted work; GWE, global work efficiency; GLS, global longitudinal strain; EF, ejection fraction; DAS28-CRP, disease activity score in 28 joints based on C-reactive protein.

Mechanistic evidence from experimental models provides a plausible pathophysiological context for these associations (58). In experimental settings, TNF-*α* exerts biphasic negative inotropic effects: acutely (within 2–5 min) via sphingomyelin-mediated inhibition of ryanodine receptor Ca^2+^ release, thereby reducing contractility and GWI; and subacutely (hours later) through iNOS induction and NO-mediated suppression of L-type calcium channels and myofilament sensitivity, leading to sustained contractile inhibition (46). TNF-α-driven upregulation of GRK2 has further been shown to desensitize *β*-adrenergic receptors in animal and *in vitro* models (47), diminishing the cardiac sympathetic response. These mechanisms offer a biologically plausible basis for the myocardial work impairment observed across disease duration strata in our cohort, though direct mechanistic evidence in human RA remains limited.

Correspondingly, experimental evidence further indicates that IL-1β and IL-6 can rapidly impair cardiomyocyte function: in vitro studies have demonstrated that IL-1β can directly inhibit L-type calcium currents in cardiomyocytes within 30 min, while IL-6 induces a concentration-dependent negative inotropic effect in human atrial muscle strips within just 2–3 min (48). More critically, these proinflammatory cytokines mediate β-adrenergic receptor desensitization in the myocardium through GRK2 upregulation, thereby impairing the cardiac response to sympathetic stimulation (47, 49). This mechanism could adversely affect myocardial work capacity under loading conditions and provides a pathophysiological context that is consistent with our observational association that overall myocardial work efficiency is negatively correlated with DAS28-CRP scores (*r* = −0.369, *p* = 0.004).

Importantly, human clinical studies provide indirect therapeutic evidence consistent with this mechanistic framework. Gerasimova et al. (9) reported in a human RA cohort that tocilizumab-mediated IL-6 suppression was associated with low cardiovascular event rates over 265 weeks, with IL-6 decreasing from 79 to 7 pg./mL (*p* < 0.05) and cardiovascular event incidence remaining low (0.53 per 100 patient-years). Systematic reviews have shown that although tocilizumab may cause a slight increase in cholesterol levels, its incidence rates of myocardial infarction (0.25 cases per 100 patient-years) and stroke (0.19 cases per 100 patient-years) remain low, indicating a favorable cardiovascular safety profile (50, 51). Similarly, Austin et al. showed that IL-6 blockade significantly modified exercise-induced cardiac adaptation compared with TNF-*α* inhibitors (52), and Park et al. (53) reported in human RA patients that elevated IL-6 correlated with LV concentric remodeling, reinforcing IL-6’s central role in RA-related myocardial changes.

Human echocardiographic studies in RA cohorts further support this association. Midtbo et al. (54) in a study of RA patients and healthy controls with preserved LVEF, demonstrated that active RA was independently associated with reduced GLS and scMWS in multivariate analysis. Their study demonstrated that patients with active RA exhibited significantly lower average stress-corrected midwall shortening (scMWS) and GLS compared to those in remission (both *p* < 0.01). Multivariate analysis further indicated that active RA was independently associated with reduced GLS (*β* = 0.21) and scMWS (β = −0.22), with both indices reflecting impaired left ventricular systolic myocardial function, independent of cardiovascular risk factors and LVEF. Similarly, Fine et al. (55) in a population sample of 87 RA patients, found significantly reduced LV and RV GLS compared with healthy controls (−15.7 ± 3.2% vs. −18.1 ± 2.4%, *p* < 0.001), with LV GLS correlating significantly with the Health Assessment Questionnaire Disability Index - a human measure of RA disease activity (−17.9 ± 4.7% vs. −20.7 ± 2.4%, *p* < 0.001). Notably, univariate regression analysis revealed a significant correlation between left ventricular GLS and the Health Assessment Questionnaire Disability Index - a measure of RA disease activity (*p* = 0.032). These findings highlight a close association between inflammatory disease activity in RA and myocardial dysfunction. Consistent with these observations, our study demonstrated a negative correlation between GWE and DAS28-CRP (*r* = −0.369, *p* = 0.004), indicating that higher disease activity is associated with lower myocardial work efficiency. These results further suggest that current disease activity may be associated with reduced myocardial work efficiency, consistent with a biological effect of inflammation on myocardial mechanics in RA patients.

In summary, disease duration provides the substrate - the “stage” - for potential myocardial vulnerability, whereas active systemic inflammation acts as the “conductor” precipitating functional decline. This pattern is consistent with findings in other systemic autoimmune diseases, in which myocardial work parameters show a stronger association with markers of disease activity than with disease duration (56). A recent review based on CMR multi-parameter imaging further emphasizes that: compared with patients with long-course SLE, patients with new-onset SLE showed more obvious myocardial edema (higher T2 value), suggesting that inflammatory activity was more active in the early stage of the disease (57). We suggest that in autoimmune conditions such as RA, proactive control of disease activity may be more critical for cardiac protection than the mere passage of diagnostic time. This distinction bears important clinical implications, supporting a strategy of active, inflammation-targeted cardioprotection for all RA patients, rather than a passive, duration-based risk stratification approach.

### 4.4 Clinical implications for cardiovascular risk monitoring in RA

The core findings of this study provide additional associative evidence suggesting that cardiovascular risk monitoring strategies in RA may warrant reconsideration to incorporate current disease activity alongside conventional duration-based assessment. These data raise the possibility that disease activity–informed risk stratification could complement traditional approaches, though prospective validation is needed before clinical implementation. Our results demonstrate that even among RA patients with preserved LVEF, myocardial work indices are significantly reduced. Notably, these changes are detectable across disease durations and show no significant correlation with disease duration, but are closely associated with current disease activity (GWE vs. DAS28-CRP: *r* = −0.369, *p* = 0.004).

Clinical translation of these findings includes:

(1) *Risk identification*: All RA patients, irrespective of duration, should undergo cardiovascular risk assessment, dispelling the misconception that short-duration RA equates to low risk.
(2) *Therapeutic timing*: For patients showing impaired MW indices—especially those with low GWE and high DAS28-CRP - clinicians should review anti-inflammatory treatment adequacy beyond joint-symptom control.
(3) *Personalized monitoring*: MW assessment should be integrated into RA follow-up programs, forming an “RA–cardiac integrated management model” based on LV-PSL technology.

By detecting subclinical myocardial impairment invisible to standard echocardiography, LV-PSL provides a sensitive, load-compensated tool for early risk identification and intervention in preserved-EF patients. Compared with GLS, MW indices possess superior diagnostic performance by accounting for afterload, enabling a forward-looking, proactive approach to cardiovascular care in inflammatory arthritis.

It should be acknowledged that widespread implementation of LV-PSL technology in routine RA management faces several practical barriers. First, myocardial work analysis requires operator proficiency in speckle-tracking echocardiography, dedicated post-processing software, and familiarity with vendor-specific platforms. Second, normative reference values for MW parameters in RA have not yet been established across populations and vendor platforms, precluding standardized threshold-based risk classification. Third, the cost-effectiveness of LV-PSL-based cardiovascular screening in RA has not been formally evaluated. In light of these constraints, we propose that LV-PSL evaluation may be most appropriately targeted, in the near term, to RA patients who meet one or more of the following criteria: (i) persistently elevated disease activity (DAS28-CRP > 3.2) despite treatment; (ii) unexplained exertional symptoms or reduced exercise tolerance; or (iii) conventional echocardiographic parameters within normal limits but with multiple cardiovascular risk factors. Universal adoption should await prospective validation of MW reference ranges and demonstration of outcome benefit in intervention trials.

### 4.5 Limitations

Several limitations warrant acknowledgment.

(1) *Sample size*: The sample size was relatively small (*N* = 94). This was largely due to our strict exclusion criteria, which prioritized a homogenous cohort free from cardiovascular confounders (such as hypertension and cardiotoxic drug history) over a larger, heterogeneous sample. Although an *a priori* sample size calculation was not performed, post-hoc power analysis yielded a value of 0.857, reflecting the observed effect size; however, the absence of an a priori calculation and the modest sample size remain important limitations that necessitate replication in larger, multi-center cohorts.
(2) *Study design*: As a cross-sectional study, the observed associations between disease activity (DAS28-CRP) and myocardial work parameters do not permit causal inferences. We cannot determine whether inflammation leads to myocardial dysfunction, whether cardiac changes influence systemic inflammation, or whether both are related to unmeasured confounding factors. The language used in the discussion interprets associations within the prevailing pathophysiological framework but acknowledges this inherent limitation of the design. Disease duration was defined based on the date of diagnosis rather than symptom onset to ensure data objectivity. We acknowledge that the potential lag time between symptom onset and diagnosis might introduce some degree of misclassification, which could theoretically dilute the effects of disease duration.
(3) *Absence of treatment exposure data*: Although patients with known cardiotoxic drug exposure were excluded, detailed data on anti-rheumatic treatment regimens - including csDMARDs, biological agents, JAK inhibitors, and corticosteroids - were not systematically collected due to the complexity of longitudinal treatment adjustments across disease durations ranging up to over three decades. This represents a meaningful limitation, as treatment exposure may independently influence both current disease activity and myocardial mechanics. Specifically, long-duration patients may have accumulated biologic exposure and achieved more effective inflammatory suppression over time, which could theoretically mask a duration-related progression of myocardial dysfunction. The comparable DAS28-CRP levels between short- and long-duration groups (Table 2, all *p* > 0.05) partially mitigate this concern, suggesting similar current inflammatory states. Nevertheless, we cannot exclude confounding by treatment history, and future studies should prospectively document treatment exposure to enable sensitivity analyses stratified by therapeutic regimen.
(4) *Lack of prognostic data*: The absence of longitudinal follow-up precludes evaluation of MW parameters as predictors of cardiovascular events and outcomes.
(5) *Myocardial work estimation*: The calculation of myocardial work relies on brachial cuff pressure as a surrogate for LV peak pressure. Although this method has been validated against invasive measurements (19), we acknowledge that it does not account for potential variations in peripheral-to-central pressure amplification, particularly in patients with increased arterial stiffness such as those with RA. This remains an inherent limitation of the current non-invasive technology.
(6) *Vendor-specific software*: Additionally, myocardial work analysis was performed using vendor-specific software (GE EchoPAC). While this platform is currently the most widely used and validated tool for non-invasive myocardial work assessment, the absolute values may not be directly interchangeable with those derived from other vendors’ software. Future efforts toward inter-vendor standardization will be essential for broader clinical applicability.
(7) *Single time-point disease activity assessment*: This study evaluated current inflammatory status using DAS28-CRP measured at the time of echocardiographic assessment. This single time-point measurement does not capture the longitudinal trajectory of disease activity, including the proportion of time spent in remission, the cumulative burden of prior high-disease-activity periods, or the pattern of therapeutic response over the disease course. As illustrated by a clinically meaningful contrast: a patient with well-controlled RA for 20 years represents a fundamentally different cumulative inflammatory exposure profile compared with a patient with continuously active disease over 5 years, yet both may display similar DAS28-CRP at assessment. Future studies incorporating longitudinal inflammatory burden metrics - analogous to HbA1c as an integrative measure in diabetes - would provide more nuanced insights into the relationship between cumulative inflammatory exposure and myocardial work impairment in RA.

## 5 Conclusion

In this study, we employed LV-PSL technology to systematically demonstrate that myocardial work impairment in RA patients is detectable across disease duration strata, including among patients with shorter disease duration, and is not restricted to those with long-standing disease. This key finding is consistent with the view that cardiac involvement in RA is not restricted to patients with long-standing disease, and that myocardial work impairment may be detectable regardless of disease duration. Instead, our results suggest that current disease activity may be more closely associated with impaired myocardial work efficiency than disease duration. These findings provide new associative insights into cardiovascular complications in RA and underscore that current disease activity, rather than cumulative disease duration, may be more closely linked to the observed myocardial work impairment.

Based on these findings and current limitations, future research should focus on:

(1) *Development of cumulative inflammatory biomarkers*: Currently, the management of RA lacks a widely accessible biomarker analogous to HbA1c in diabetes, which can quantitatively reflect average disease activity over a prolonged period. Future studies should aim to identify such longitudinal markers to better capture the cumulative inflammatory burden contributing to myocardial remodeling, rather than relying solely on single-point measures like DAS28-CRP.
(2) *Large-scale prospective cohorts*: To validate MW parameters as independent predictors of cardiovascular outcomes and to establish standardized reference ranges for the RA population.
(3) *Mechanistic integration*: Studies combining cytokine profiling and multimodality imaging are needed to elucidate the molecular mechanisms driving these early functional changes and to assess their reversibility.
(4) *Therapeutic intervention trials*: Assessing whether aggressive anti-inflammatory strategies (e.g., biologic agents, JAK inhibitors) can reverse subclinical MW abnormalities.
(5) *Clinical standardization*: Establishing unified LV-PSL protocols, quality-control systems, and collaborative management algorithms between rheumatology and cardiology.

In conclusion, this study lays the foundation for developing inflammation-targeted cardiac protection strategies in RA. LV-PSL technology emerges as a promising tool for cardiovascular risk stratification and therapeutic monitoring, with the potential to enhance long-term outcomes in this high-risk population.

## Data Availability

The original contributions presented in the study are included in the article/Supplementary material, further inquiries can be directed to the corresponding author.

## Supplementary material

SUPPLEMENTARY FIGURE 1 Bland-Altman plots for intra- and inter-observer reproducibility of GWI and GLS. **(A)** Intra-observer and **(B)** inter-observer agreement for GWI. **(C)** Intra-observer and **(D)** inter-observer agreement for GLS. The solid horizontal lines represent the mean difference (bias) between measurements, while the dashed lines represent the 95% limits of agreement (mean difference ± 1.96 SD). GWI, Global Work Index; GLS, Global Longitudinal Strain; SD, Standard Deviation.

## Glossary

RA: Rheumatoid arthritis
LVEDV: Left ventricular end-diastolic volume
CV: Cardiovascular
LVESV: Left ventricular end-systolic volume
ESC: European Society of Cardiology
GCW: Global Constructive Work
TNF-*α*: Tumor necrosis factor-α
GWW: Global Wasted Work
IL-1β: Interleukin-1β
GWE: Global work efficiency
IL-6: interleukin-6
ANOVA: Analysis of variance
LVEF: Left ventricular ejection fraction
HSD: Honest significant difference
MW: Myocardial work
ICC: Intraclass correlation coefficient
2D-STE: Two-dimensional speckle-tracking echocardiography
RF: Rheumatoid factor
GLS: Global longitudinal strain
CRP: C-reactive protein
GWI: Global Work Index
ESR: Erythrocyte sedimentation rate
LV-PSL: Left ventricular pressure–strain loop
NEUT: Neutrophil percentage
WHO: World Health Organization
HR: Heart rate
OGTT: Oral glucose tolerance test
PSL: Pressure-strain loop
ISH: International Society of Hypertension
LV: Left ventricular
LVEDD: Left ventricular end-diastolic diameter
STE: Speckle-tracking echocardiography
IVSD: Interventricular septal thickness at end-diastole
SLE: Systemic lupus erythematosus
LVPWD: Left ventricular posterior wall thickness at end-diastole
scMWS: Stress-corrected midwall shortening
