# Supplementary figures and images for: Myocardial work abnormalities in rheumatoid arthritis with preserved left ventricular ejection fraction are more closely related to disease activity than to disease duration: a prospective cross-sectional study
Source: Front Med (Lausanne). 2026 Jul 6;13:1872016. doi: 10.3389/fmed.2026.1872016 (PMC13381644; doi:10.3389/fmed.2026.1872016)

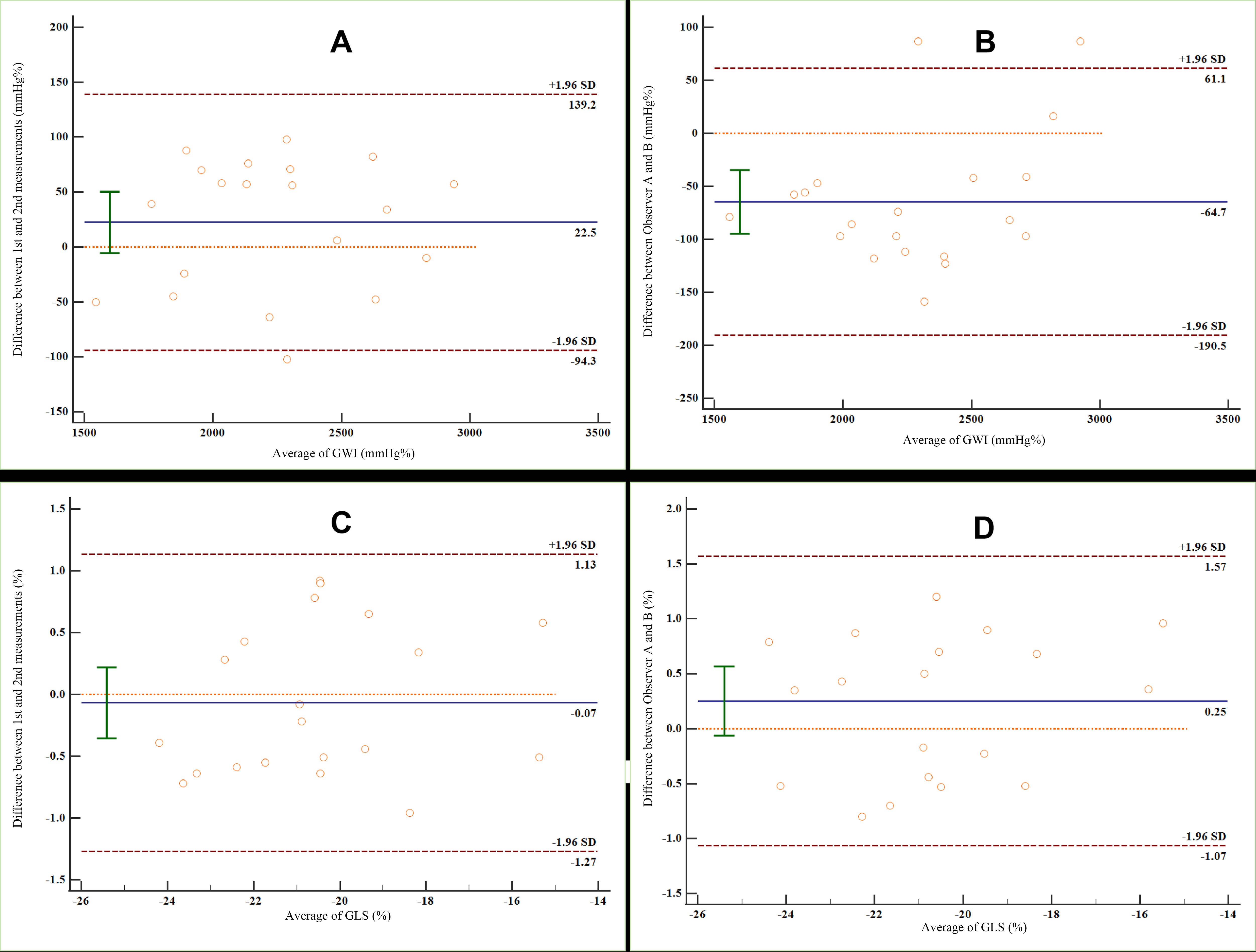

Supplement: SUPPLEMENTARY FIGURE 1 — Bland-Altman plots for intra- and inter-observer reproducibility of GWI and GLS. (A) Intra-observer and (B) inter-observer agreement for GWI. (C) Intra-observer and (D) inter-observer agreement for GLS. The solid horizontal lines represent the mean difference (bias) between measurements, while the dashed lines represent the 95% limits of agreement (mean difference ± 1.96 SD). GWI, Global Work Index; GLS, Global Longitudinal Strain; SD, Standard Deviation. [file Supplementary_Figure_1.TIF]
